# Intravital Microscopy Evidence That Methylene Blue Should Be a Vasopressor-Sparing Agent in Sepsis Vasoplegia

**DOI:** 10.21470/1678-9741-2023-0066

**Published:** 2024-03-20

**Authors:** Fabiola Leslie Antunes Cardoso Mestriner, Pedro Brüch Dantas, Jéssyca Michelon Barbosa, Paulo Roberto B. Evora, Christiane Becari

**Affiliations:** 1 Department of Surgery and Anatomy, Faculdade de Medicina de Ribeirão Preto, Universidade de São Paulo, Ribeirão Preto, São Paulo, Brazil; 2 Department of Biological Sciences, Faculdade de Odontologia de Bauru, Universidade de São Paulo, Bauru, São Paulo, Brazil

**Keywords:** Methylene Blue, Microcirculation, Microvessels, Endothelium, Lipopolysaccharides

## Abstract

Microvasculature failure is expected in sepsis and at higher amine concentrations. Therefore, special attention focused individually on microcirculation is needed. Here, we present that methylene blue can prevent leukocytes from adhering to the endothelium in a rat model of lipopolysaccharide-induced endotoxemia. As hypothesis evidence, an intravital microscopy image is presented.

## INTRODUCTION

**Table:** 

Abbreviations, Acronyms & Symbols	
**LPS**	= Lipopolysaccharide
**MB**	**= Methylene blue**

Gomes^[1]^ first described vasoplegic syndrome in heart surgery patients have been undergoing treatment with methylene blue (MB) for it. Evora et al (1996)^[2]^ were the ones who suggested the use of MB for treatment. In 1996, Andrade et al.^[3]^ first documented this therapeutic approach in heart surgery patients. MB successfully treats vasopressor-refractory septic shock vasoplegia by inhibiting endothelial nitric oxide and improving responsiveness to amines. However, only one relevant study has explored the microcirculatory effects of MB^[4]^.

### Intravital Microscopy Method

**Fig. 1:**
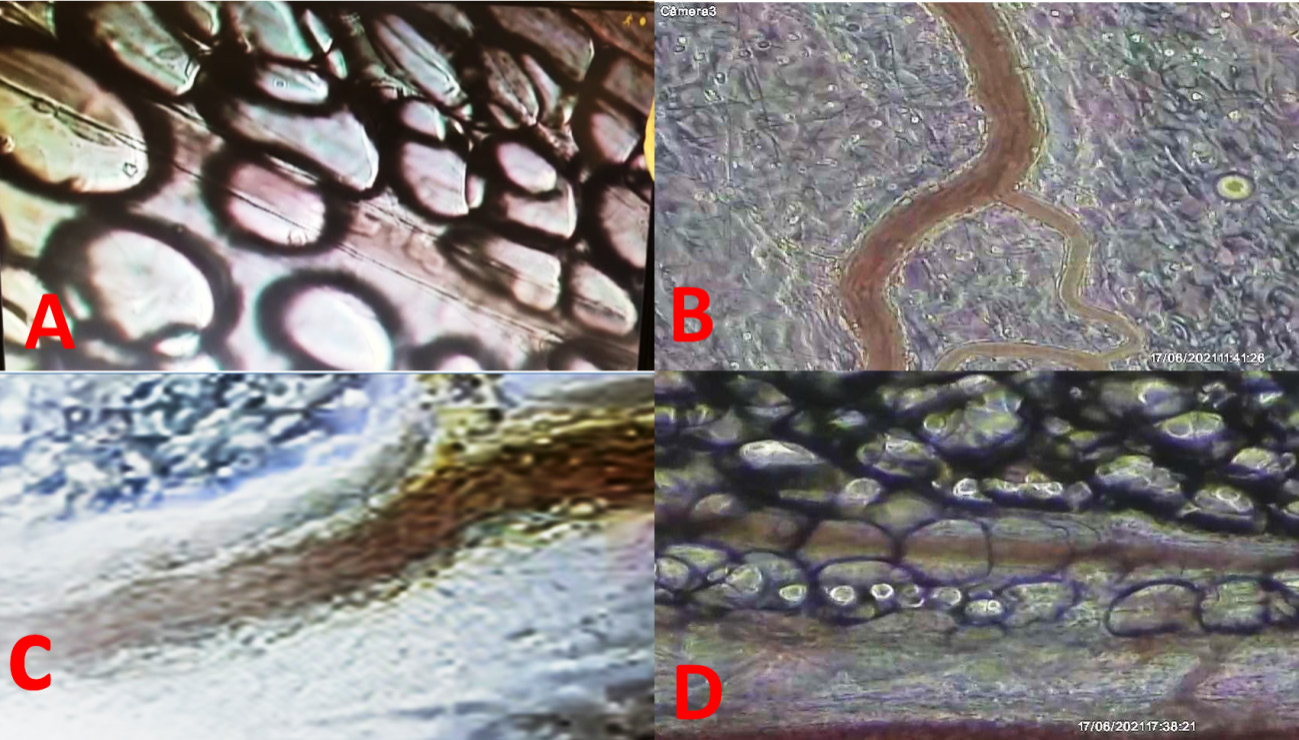
Methylene blue (MB) inhibiting neutrophil leukocyte rolling/adhesion after lipopolysaccharide (LPS) intravenous dose. (A) Postcapillary venules of saline group (saline at zero and 15 min); (B) postcapillary venules of LPS group (LPS at zero and saline at 15 min); (C) postcapillary venules of only MB (MB at zero and saline at 15 min); (D) postcapillary venules of MB after LPS doses (LPS at zero and MB at 15 min). # Intra peritoneal image of postcapillary venules. (A) Control (roller mean = 52.4, adhesion mean = 1.3); (B) LPS + salina (roller mean = 816, adhesion mean = 9.4); (C) LPS + MB (roller mean = 28.1, adhesion mean = 3.2); (D) MB + LPS (roller mean = 76.4, adhesion mean = .6).

**Fig. 2:**
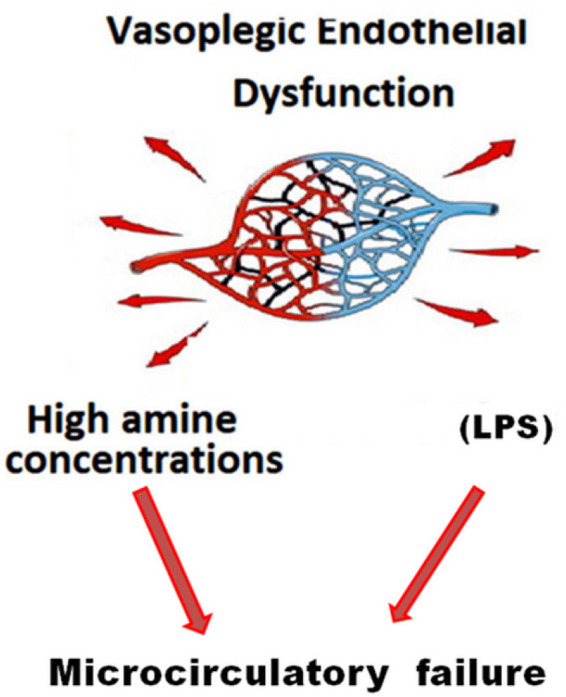
Schematic representation of microcirculatory damage consequent to lipopolysaccharide (LPS) and high doses of amine.

This study involved adult male Hannover rats under controlled conditions approved by the Committee on Ethics in Animal Experimentation of the Faculdade de Medicina de Ribeirão Preto, Universidade de São Paulo (2/2015). We anesthetized the animals, exteriorized the mesentery, and examined postcapillary venules with diameters of 10-18 micrometers. We evaluated leukocytes adhering to the endothelium within 10-micrometer venule lengths. Furthermore, we considered leukocytes adhered for up to 30 seconds for the microcirculatory protective effect (Figures 1 and 2).

The technique is particularly useful for studying microcirculation. It allows researchers to observe blood flow through capillaries and venules, providing insights into blood perfusion, oxygen exchange, and interactions between blood cells and vessel walls.

## COMMENTS

We state that the medical literature underestimates the importance of cyclic guanosine monophosphate. Combining three concepts could yield better results against high mortality rates in critically ill patients: (1) using “broad-spectrum vasopressors”, (2) employing vasopressor-sparing strategies, and (3) protecting microcirculation.

MB should serve as a vasopressor-sparing agent. We need progressively minor concentrations of amines to maintain blood pressure (around 65 mmHg). Additionally, using high amine concentrations independently of blood pressure becomes reasonable. We anticipate the disclosure, hoping that new research groups interested in the subject will emerge. Therefore, we firmly believe that the briefly discussed concepts will incorporate as paradigms in vasodilatory shock treatment.

“Microcirculatory protection” is an old concept. It assumes that microvasculature failure is unavoidable even with arterial pressure under control, with increasing amine concentrations. Therefore, special attention focused individually on microcirculation is needed. The image presented, corresponding to the record of an experiment, has been checked and reproduced in our laboratory. With the hope of motivating different research groups, we decided the hypothesis disclosure. Chances are that the briefly discussed hypothetical concepts can be incorporated as paradigms in treating vasodilatory shock^[5-7]^.

**Table:** 

Author's Roles & Responsibilities	
FLACM	Substantial contributions to the conception or design of the work; or the acquisition, analysis, or interpretation of data for the work; drafting the work or revising it critically for important intellectual content; final approval of the version to be published; agreement to be accountable for all aspects of the work in ensuring that questions related to the accuracy or integrity of any part of the work are appropriately investigated and resolved
PBD	Substantial contributions to the conception or design of the work; or the acquisition, analysis, or interpretation of data for the work
JMB	Substantial contributions to the conception or design of the work; or the acquisition, analysis, or interpretation of data for the work
PRBE	Substantial contributions to the conception or design of the work; or the acquisition, analysis, or interpretation of data for the work; drafting the work or revising it critically for important intellectual content; final approval of the version to be published; agreement to be accountable for all aspects of the work in ensuring that questions related to the accuracy or integrity of any part of the work are appropriately investigated and resolved
CB	Substantial contributions to the conception or design of the work; or the acquisition, analysis, or interpretation of data for the work; drafting the work or revising it critically for important intellectual content; final approval of the version to be published; agreement to be accountable for all aspects of the work in ensuring that questions related to the accuracy or integrity of any part of the work are appropriately investigated and resolved
